# Transcriptional responses and secondary metabolites variation of tomato plant in response to tobacco mosaic virus infestation

**DOI:** 10.1038/s41598-024-69492-3

**Published:** 2024-08-22

**Authors:** Mona Rabie, Dalia G. Aseel, Hosny A. Younes, Said I. Behiry, Ahmed Abdelkhalek

**Affiliations:** 1 Department of Botany and Microbiology, Faculty of Science, Alexandria University, Alexandria, 21511 Egypt; 2 Plant Protection and Biomolecular Diagnosis Department, Arid Lands Cultivation Research Institute, City of Scientific Research and Technological Applications, Alexandria, 21934 Egypt; 3 Agricultural Botany Department, Faculty of Agriculture (Saba Basha), Alexandria University, Alexandria, 21531 Egypt

**Keywords:** Tomato, Tobacco mosaic virus, Gene expression, Polyphenolic compounds, HPLC, GC–MS, Virology, Plant immunity, Plant molecular biology, Plant stress responses, Secondary metabolism

## Abstract

The present study focused on the impact of infection with the tobacco mosaic virus (TMV). Specifically, changes in phytochemicals and gene activity related to pathogenesis-related and phenylpropanoid pathway genes in tomato plants (*Solanum lycopersicum* L.) during a period of 2–14 days post-inoculation (dpi). According to TEM investigation and coat protein sequence analysis, the purified TMV Egyptian AM isolate (PP133743) has a rod-shaped structure with a diameter of around 110 nm. The RT-qPCR analysis revealed that *PR-1* showed an initial increase after TMV infection, as seen in the time-course analysis. In contrast, *PR-2* was consistently elevated throughout the infection, suggesting a stronger reaction to the virus and suppressing *PAL* expression at 6 to 14 dpi. The expression levels of *HQT* and *CHS* transcripts exhibited alternating patterns of up-regulation and down-regulation at different time intervals. The HPLC and GC–MS analysis of control- and TMV-infected tomato extracts revealed that different phenolic, flavonoid, and fatty acid compounds were increased (such as naringenin, rutin, flavone, ferulic acid, and pyrogallol) or significantly decreased (such as salicylic acid and chlorogenic acid) after TMV infection. The ability of TMV to inhibit most polyphenolic compounds could potentially accelerate the viral life cycle. Consequently, focusing on enhancing the levels of such suppressed compounds may be critical for developing plant viral infection management strategies.

## Introduction

Plants are regarded as passive subjects that are exposed to biotic (viroids, viruses, bacteria, fungi, nematodes, or insects) and abiotic (salinity and drought) stimuli. As a result, they have developed a wide range of complex defensive systems to survive various types of stress and have a significant resistance impact in plants^1–3^. Plant viral infections present a substantial threat to plant biosecurity, leading to extensive crop damage worldwide^4^. Because of its wide host range (about 66 families with more than 900 plant species) and devastating infection outcomes, tobacco mosaic virus (TMV, genus Tobamovirus) ranks among the most infectious plant viruses^5^. TMV can be mechanically transmitted through direct physical contact with infected plants, contaminated agricultural tools, or contaminated seeds^6^. Furthermore, TMV particles are highly stable, allowing them to remain viable for extended periods in infected substances like decomposing leaves or soil, retaining the ability to cause reinfection^7^. Tomatoes (*Solanum lycopersicum* L.), the second most cultivated and consumed vegetable crop worldwide, are commonly used as model organisms in scientific research because of their amenability to genetic alteration^8^. They are utilized to investigate metabolic and fundamental biological processes as well as to analyze interactions between plants and pathogens^9^. Systemic leaf mosaic, necrosis, and leaf chlorosis are some of the morphological defects caused by TMV infection^10^. In addition, a more severe case of TMV infection can lead to systemic changes in the organs responsible for flowering, which in turn can postpone fruit ripening, decrease crop yield, and eventually cause crop failure^11^.

Plant secondary metabolites aid in plant survival under challenging conditions and provide resistance against many biological and environmental stressors^12^. The generation of various natural chemicals, including flavonoids, phenols, and polyphenols, as well as important biomolecules like carbohydrates and chlorophyll and some antioxidant enzymes like POX, PPO, APX, and CAT, is one of the most well-established defensive mechanisms^13–15^. The response to pathogens, both incompatible and compatible interactions, exhibits comparable properties, such as inducing plant signaling molecules like salicylic acid and ethylene, as well as producing pathogenesis-related (PR) proteins^1617^. Plant viral infection alters polyphenolic contents by modulating the levels of phenolic compounds and antioxidative enzymes. These changes result in cell damage, the generation of reactive oxidant species (ROS), and the initiation of pathogen-triggered defensive responses^18^. Biochemical defense processes including PRs proteins, phenylalanine ammonia-lyase, and ROS improve plant defense by producing secondary bioactive compounds^19^. Moreover, the viral infection was discovered to affect the host's gene expression pattern and responses, with gene silencing possibly associated with antiviral systems^4^. The majority of research on plant-pathogen interactions has focused on plant-bacterial and plant-fungal interactions, whereas plant reactions to viruses are less well understood. The exact mechanisms of TMV-host interactions are not yet fully understood.

To further investigate and clarify the interactions between TMV and tomato plants, we first isolated, purified, characterized, and molecularly identified an Egyptian TMV isolate. Under greenhouse conditions, the expression of various defense-related genes at 2, 6, 10, and 14 days post-TMV inoculation (dpi) was evaluated. These genes included three polyphenolic pathway genes phenylalanine ammonia-lyase (*PAL*), hydroxycinnamoyl Co A: quinate hydroxycinnamoyl transferase (*HQT*), chalcone synthase (*CHS*) and two pathogenesis-related genes (*PR-1* and *PR-2*). Furthermore, high-performance liquid chromatography (HPLC) analysis of polyphenolic (phenolic and flavonoid) compounds was carried out at the early stage of infection at 6 and 14 dpi. Gas chromatography-mass spectrometry (GC–MS) was also used to identify several chemical compounds that either gathered or decreased in tomato leaves after being infected with TMV at 6 and 14 dpi.

## Results

### TMV isolation, purification and biomolecular characterization

**Figure 1:**
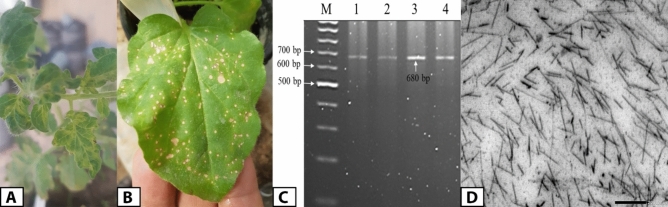
Tomato (*Solanum lycopersicum L*.) leaves mosaic pattern, leaf malformation and chlorosis symptoms after infection by TMV at 14 dpi (**A**). Single local lesions of TMV-infected *Nicotiana glutinosa* leaves at 5 dpi (**B**). RT-PCR amplification of *TMV-CP* gene (**C**). Transmission electron microscopy of rod shaped purified TMV particles about 18 nm in diameter (**D**). The complete gel image is presented in supplementary file (Figure S1).

**Figure 2:**
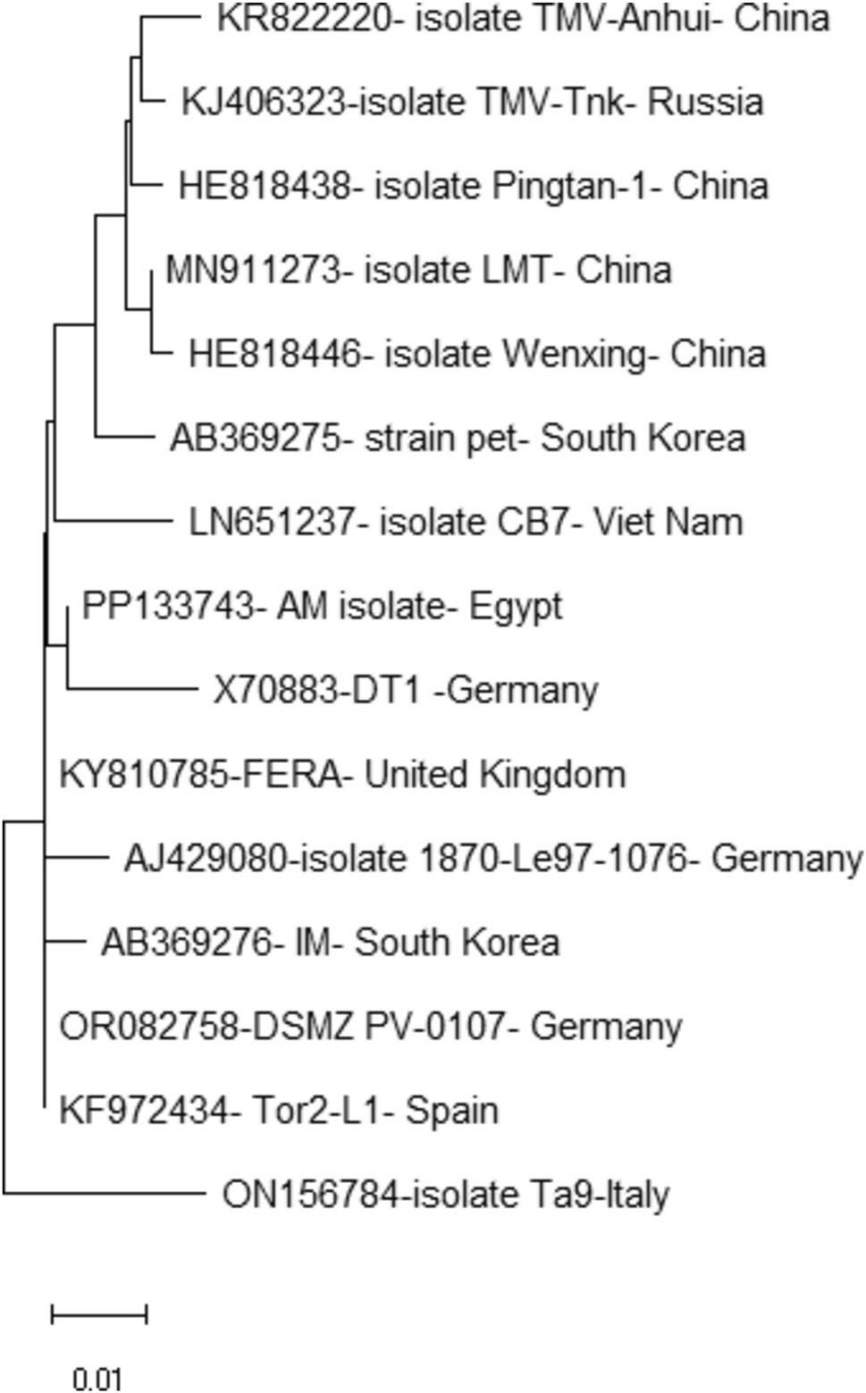
Neighbor-joining phylogenetic trees of an Egyptian TMV isolate (PP133743) based on the nucleotide sequences of the *CP* gene compared to some other previously reported TMV isolates.

The open field collected tomato samples displayed distorted leaves, blistering, mosaic patterns, and chlorotic lesions, which are typical TMV systemic symptoms compared to healthy ones (Fig. 1A). Using a DAS-ELISA test, almost 65% of the samples tested positive for TMV. At 4 to 5 dpi, a pure TMV strain was isolated from a single local lesion on *Nicotiana glutinosa* leaves (Fig. 1B). Subsequently, RT-PCR using a *TMV-CP* gene-specific primer resulted in the amplification of 680 bp (Fig. 1C). TEM examination revealed that the purified TMV particles had a rod-shaped structure with a diameter of approximately 110 nm (Fig. 1D). The purified viral concentration was around 4.6 mg per 100 g of fresh leaf mass. After purifying the PCR product, the amplicon was sequenced and submitted to GenBank with the accession number PP133743. The phylogenetic tree indicated that the TMV Egyptian AM isolate shared 99% similarity with strain TD1 from Germany (Fig. 2).

### Sample collection and virus detection

**Figure 3:**
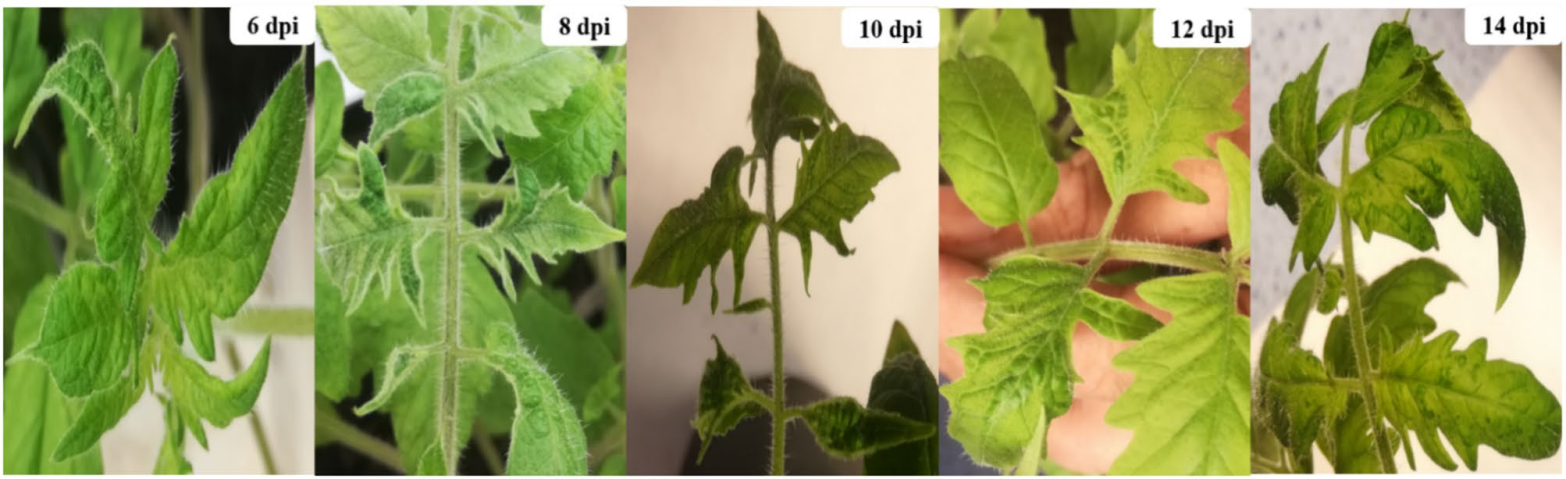
The development of TMV symptoms on inoculated tomato plants at different time intervals. The mosaic symptoms started to develop at 8 dpi and were clearly visible at 12 dpi. At 14 dpi, a severe mosaic with chlorosis symptoms was observed.

Under greenhouse conditions, TMV-infected tomato plants displayed multiple morphological changes that induced a systemic mosaic with chlorosis of tomato leaves similar to those reported on naturally infected plants. Mechanically infected tomato plants started to develop symptoms at 8 dpi, with distinct and easily noticeable symptoms at 12 dpi and clearly severe symptoms reported at 14 dpi (Fig. 3). The tomato leaves were gathered at 2, 4, 6, 8, 10, 12, and 14 dpi for each treatment as well as mock-inoculated plants. The I-ELISA data (Table 1) revealed that the first detection of viral infections was at 6 dpi with an ELISA value of 0.424, compared to the control with an ELISA value of 0.111. Then, there was a dramatic increase in the accumulation levels, reaching a maximum of 1.174 at 14 dpi. No virus was detected in mock-inoculated samples.

**Table 1:** I-ELISA detection of TMV in 1:10 dilution of sap extracted of inoculated tomato plants collected at different time intervals.

Days post inoculation (dpi)	Absorbance ELISA values at 405 nm		
	Infected (EV ± SD)	Mock (EV ± SD)	Result
2	0.203 ± 0.045	0.110 ± 0.096	-
4	0.212 ± 0.104	0.110 ± 0.046	-
6	0.424 ± 0.109	0.111 ± 0.074	+
8	0.580 ± 0.176	0.124 ± 0.056	+
10	0.750 ± 0.132	0.152 ± 0.087	+
12	0.926 ± 0.138	0.165 ± 0.036	+
14	1.174 ± 0.176	0.227 ± 0.032	+

The ELISA value that equal double of mock (healthy control) was considered as positive.

*EV* ELISA value, *SD* standard deviation.

+ : Positive; − : Negative.

### Expression of defense-responses genes over time using RT-qPCR

**Figure 4:**
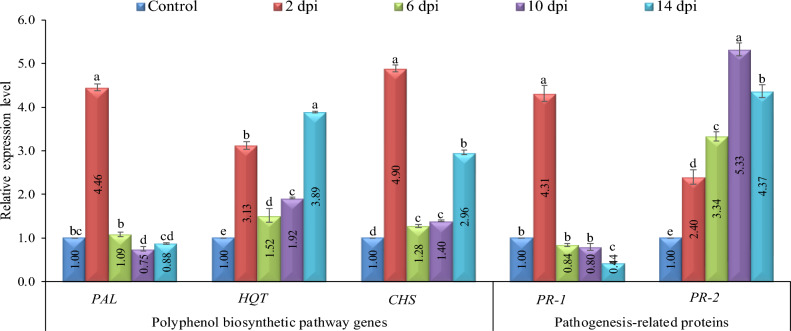
The relative transcriptional levels of *PAL*, *HQT*, *CHS*, *PR-1*, and, *PR-2* genes at 2, 6, 10 and 14 dpi of TMV compared to control. Each column showed the average of three biological replicates. Small letters indicate statistically significant differences between samples. The values in columns that share the same letter don't vary considerably.

The RT-qPCR results demonstrated that *PR-1* was induced after TMV infection of tomato plants (Fig. 4). At 2 dpi, the expression level increased rapidly, reaching 4.31-fold higher than the control. Subsequently, it significantly decreased between 6 and 14 dpi and showed relative transcript levels of 0.84-fold to 0.44-fold, respectively, lower than the control. This decline may be attributed to TMV's capacity to suppress plant defense mechanisms. Regarding *PR-2*, its expression exhibited a substantial increase over the entire duration, indicating a more significant response to viral infection compared to the control (Fig. 4). By 2 dpi, it showed rapid activation, exhibiting a 2.4-fold rise in relative expression compared to the control. The transcription level increased to 3.34-fold at 6 dpi and 5.33-fold at 10 dpi. At 14 dpi, there was a slight decrease in *PR-2* expression, with a relative expression level of 4.37-fold higher than the control. The results showed that TMV consistently activated *PR-2* in infected tomato tissues at all time intervals studied (Fig. 4).

The *PAL* gene exhibited a rapid induction in response to TMV infection, reaching an expression level 4.46-fold higher than the mock treatment at 2 dpi (Fig. 4). However, the expression level decreased rapidly, reaching its lowest point at 10 dpi, with a relative transcription level 0.75-fold lower than the control. At 14 dpi, the expression level was slightly raised, reaching 0.88 -fold. At 2 dpi, there was an initial increase in the expression of the *HQT* gene, which encodes for hydroxycinnamoyl CoA quinate transferase. The infected plants exhibited a relative expression level that was 3.13 times greater than that of the control plants. At 6 dpi, *HQT* expression decreased to 1.52-fold. Nevertheless, at 10 dpi, it exhibited another rise, reaching a peak transcriptional level that was 3.89 times higher than that of the control plants (Fig. 4). At 2 dpi, CHS had a 4.9-fold higher relative expression level than the control. CHS expression was then lowered to relative transcript levels 1.28 and 1.4-fold greater than the control at 6 and 10 dpi. Nonetheless, by 14 dpi, transcription levels were 2.96 times higher than in the control group (Fig. 4).

### HPLC analysis of phenolic compounds in ethanolic tomato extracts

**Figure 5:**
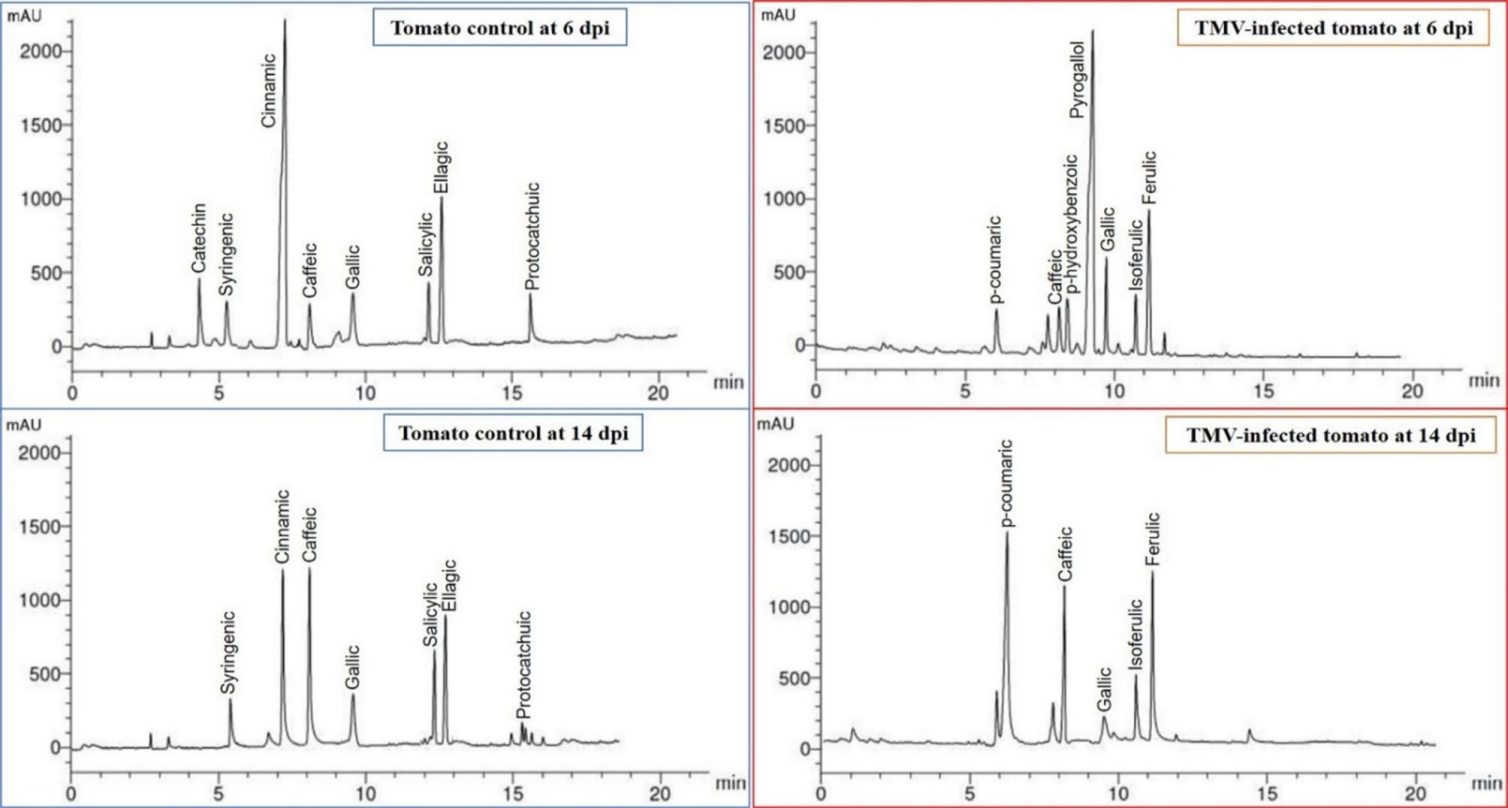
HPLC chromatography of the detected phenolic compounds in an ethanolic tomato plant extract. Mock-inoculated plants (tomato control at 6 and 14 dpi) and TMV-infected plants at 6 dpi and 14 dpi.

**Figure 6:**
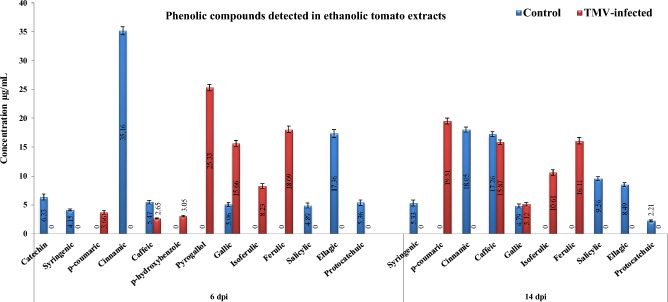
The level of detected phenolic compounds in the ethanolic extract of mock- and TMV-inoculated tomato plants at 6 and 14 dpi. Each column showed the average of three biological replicates.

Figures 5 and 6 showed phenolic compounds in ethanolic extracts of tomato plants at 6 and 14 dpi. The control group had syringenic, cinnamic, caffeic, gallic, salicylic, ellagic acid, and protocatchuic phenolic compounds at 6 and 14 dpi (Fig. 6). In contrast, one additional compound, catechin, was detected in tomato control at 6 dpi with a concentration of 6.33 μg/mL. Upon TMV inoculation (Fig. 5), three phenolic compounds (*p*-coumaric, isoferulic, and ferulic) were induced at 6 and 14 dpi. At 6 dpi, *p*-bydroxybenzoic and pyrogallol were induced in TMV-infected tissues. Two compounds, cinnamic (35.16 μg/mL) and ellagic acid (17.36 μg/mL), demonstrated the greatest levels in mock-treated tomato extracts at 6 dpi. While the greatest levels of cinnamic (18.05 μg/mL) and caffeic (17.26 μg/ml) were shown at 14 dpi, The highest levels of pyrogallol (25.33 μg/mL) and ferulic acid (18.09 μg/mL) were detected in TMV-infected plants at 6 dpi. Also, *p*-bydroxybenzoic was detected at a low concentration (3.05 μg/mL) at 6 dpi only (Fig. 5).

### HPLC analysis of flavonoid compounds in ethanolic tomato extracts

**Figure 7:**
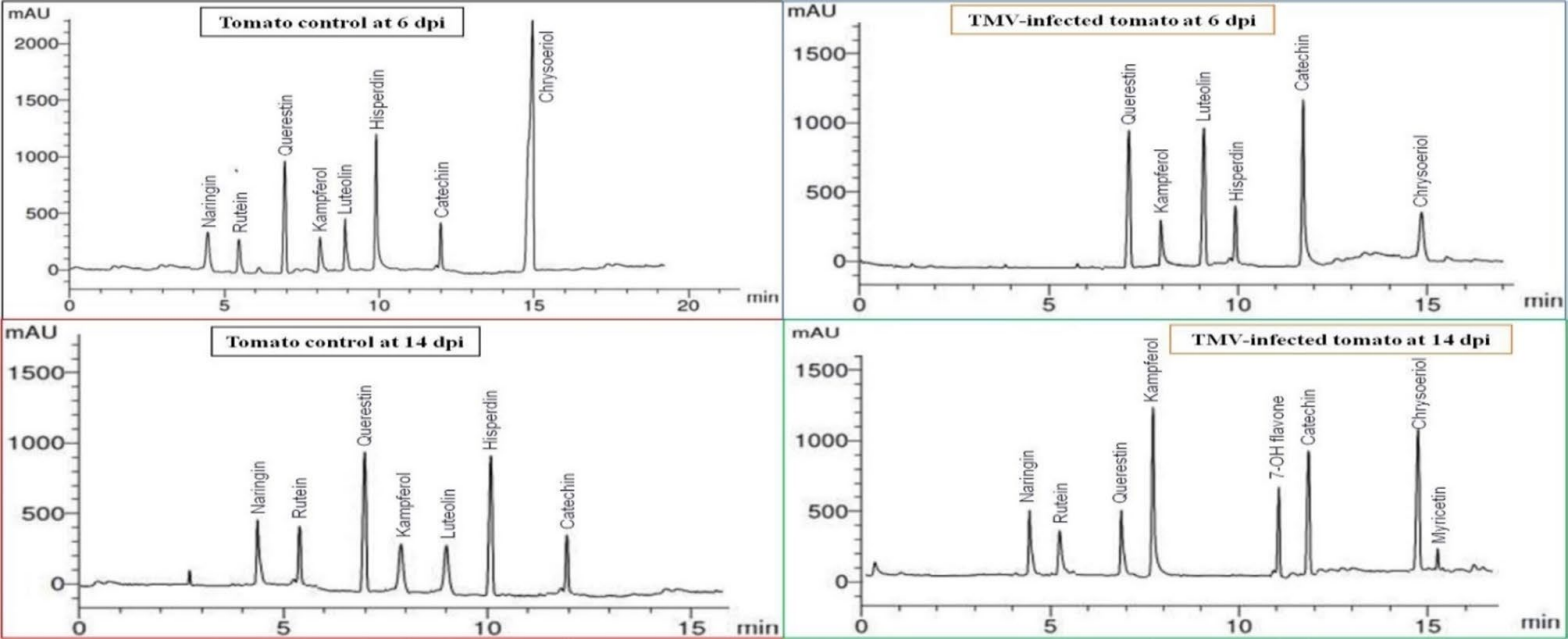
HPLC chromatography of the detected flavonoid compounds in an ethanolic tomato plant extract. Mock-inoculated plants (tomato control at 6 and 14 dpi) and TMV-infected plants at 6 dpi and 14 dpi.

**Figure 8:**
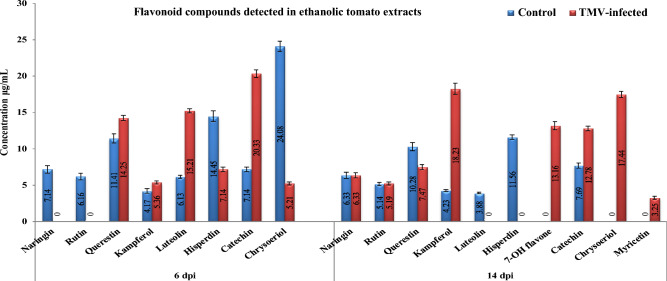
The level of detected flavonoid compounds in the ethanolic extract of mock- and TMV-inoculated tomato plants at 6 and 14 dpi. Each column showed the average of three biological replicates.

At 6 dpi, six flavonoid compounds were found in both mock- and TMV-inoculated tomato plants. However, only the control plants had naringin (7.14 μg/mL) and rutin (6.16 μg/mL) (Fig. 7). The mock treatment exhibited the most significant levels of querestin and hisperdin, measuring 14.41 and 14.45 μg/mL, respectively, at 6 dpi. At 14 dpi, the levels were 10.28 and 11.56 μg/mL, respectively (Fig. 8). Additionally, chrysoeriol was detected at a concentration of 24.08 μg/mL at 6 dpi. The highest amounts of luteolin and catechin were found in TMV-infected plants at 6 dpi, with values of 15.21 and 20.33 μg/mL, respectively (Fig. 8). However, myricetin is induced in response to TMV infection at a low concentration of 3.25 μg/mL at 14 dpi. Kampferol and chrysoeriol had the highest concentrations in TMV-infected extracts, at 18.23 and 17.44 g/mL, respectively.

### GC–MS analysis of ethanolic tomato extracts

**Figure 9:**
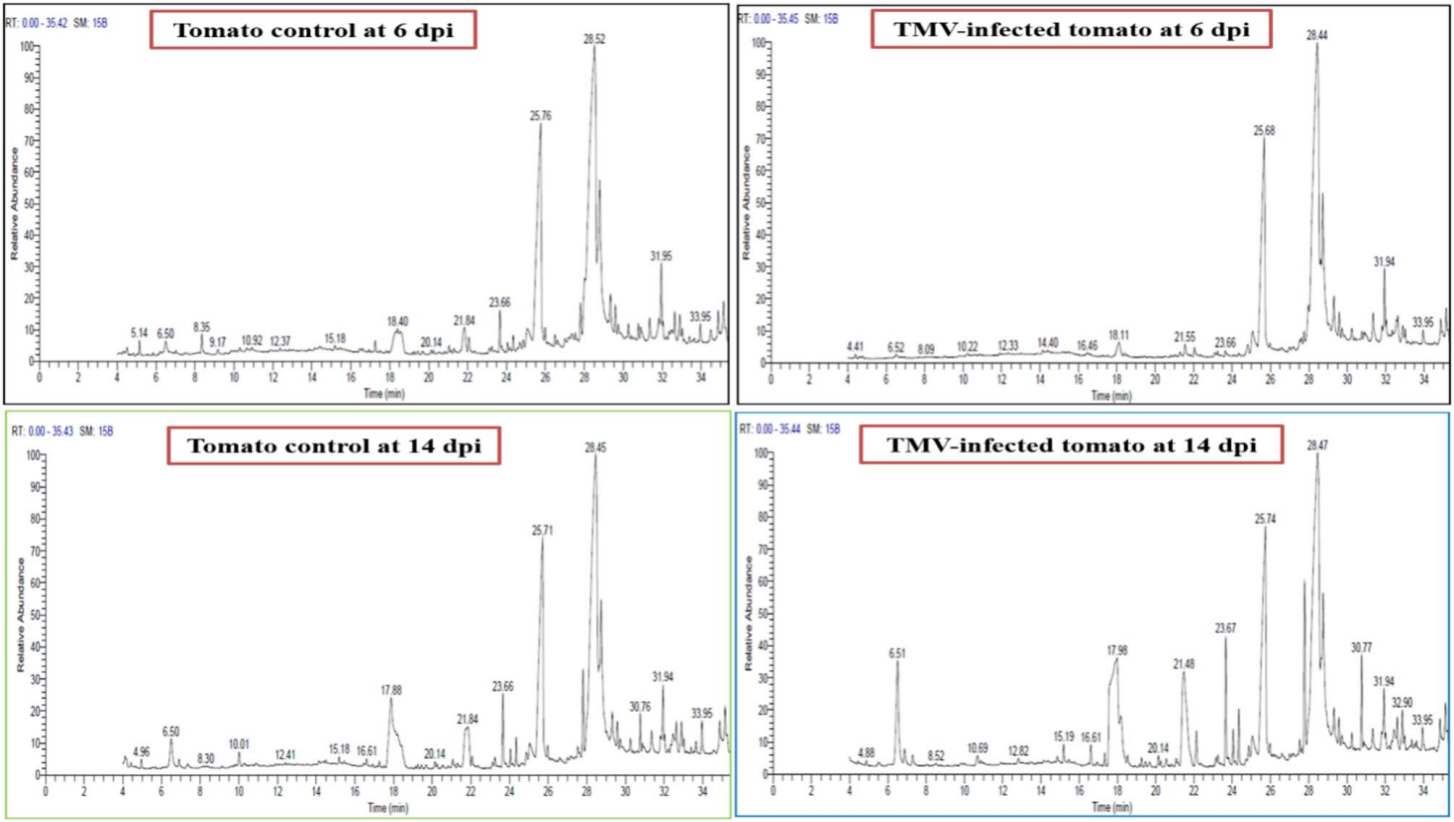
Chromatograms of ethanolic extract of tomato control plant and TMV-infected plants at 6 and 14 dpi, analyzed by GC–MS.

**Figure 10:**
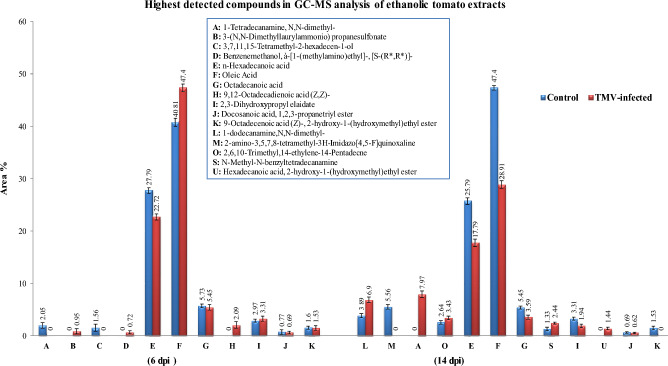
Comparing the most detectable compounds and their area percentage (shown above the columns) in the ethanolic extract of tomato control and TMV-infected plants at 6 and 14 dpi. Each column showed the average of three biological replicates.

The GC–MS analysis identified 17 compounds in the tomato extract, with 11 of them being recognized at a concentration peak of 6 dpi. At 6 dpi, a total of six distinct compounds were detected in both the mock-infected and TMV-infected plants. The compounds that were found were n-hexadecanoic acid, oleic acid (which had the highest concentration), octadecanoic acid, 2,3-dihydroxy propyl elaidate, docosanoic acid, 1,2,3 propanetriyle ester, and 9-octadecenoic acid (Z)-,2-hydroxy-1-(hydroxyl methyl) ethyl ester (Figs. 9 and 10). However, 3-(N, N-dimethylaurylammonio) prpanesulfonate, benzene, and 9,12-octadecadienic acid (Z,Z) were only detected in infected tomatoes at 6 dpi. Otherwise, 1-tetradecanamine, N, N-diethyle, and 3,7,11,15-tetramethyle 1–2-hexadecen-1-ol were only found in tomato control plants at 6 dpi (Fig. 9). At 14 dpi, 12 compounds were detected. At 14 dpi, the compound 2-amino-3,4,7,8-tetramethyl-3H-imidazo was detected only in mock-inoculated plants. At 14 days post-infection, three chemicals—1-dodecanamine, N, N-dimethyle, 2,6,10-trimethyl,14-ethylene-14-pentadecene, and N-methyl-N-benzyltetradecanamine—were only found in tomato plants that had been infected with TMV or a fake virus. At 6 dpi, only 1-tetradecanamine,N,N-diethyle, was found in tomato control plants. 3-(N,N-dimethylaurylammonio) prpanesulfonate, benzene, 9,12-octadecadienic acid (Z, Z) were detected in TMV-inoculated tomato extracts at 6 dpi. At 14 dpi, the levels of these compounds significantly decreased in both mock-inoculated plants and TMV-inoculated tomato extracts.

## Discussion

The tobacco mosaic virus causes devastating crop losses in numerous ornamental and vegetable crops across the world. Following viral infection, numerous features and alterations in gene expression levels involving stress and defense responses have been documented^20^. The present investigation involved the identification, purification, and molecular characterization of the TMV isolate AM (Acc# PP133743) through analysis of the CP gene sequence. Inoculated tomato plants exhibited expected symptoms such as chlorosis, leaf deformation, and mosaic patterns when inoculated with TMV isolate. TEM and DAS-ELISA experiments supported the identification of this virus isolate as TMV. Regarding this, TMV levels tested by I-ELISA were found to rise, in particular and considerably, at 6 and 14 dpi. This study focused on the phytochemical alterations that TMV infection causes in tomato plants. In fact, plants exhibit a considerable resistance effect due to phenolic compounds^3^. Our goal was to clarify how tomato plants growing in greenhouses respond to exogenously administered TMV in terms of defense mechanisms. Tomato tissues resist TMV invasion by accumulating various phenolic chemicals and activating defense-related genes, including those that encode PR proteins^21^.

In this regard, the RT-qPCR results of this study revealed that, once TMV had infected the plants, PR-1 was quickly increased in tomatoes. *PR-1* is thought to be a critical regulator of systemic acquired resistance (SAR) and may indicate early defense responses in plants^22^. Furthermore, salicylic acid (SA) significantly contributes to plant immunity and is an essential signal phytohormone molecule of SAR in plants. In addition, its expression is linked to the induction of the *PR-1* gene^17^. According to the expression profile found in the time-course investigation, *PR-1* is rapidly and early induced after TMV infection of tomatoes. This finding was consistent with several investigations that found a link between early defense responses and the activation of *PR-1*^23^. At 6 to 14 days after infection with TMV, the expression of *PR-1* gene decreased. This was linked to the lack of SA detected by HPLC at 6 to 14 dpi. Thus, spraying SA at the initial stages of infection may help plants develop viral resistance. The expression levels of the *PR-2* gene were up-regulated following TMV infection. *PR-2*, which encodes 1,3-glucanases, limits callose deposition close to plasmodesmata (PD), thereby facilitating communication between cells and long-range signaling^24^. It was proven that TMV increases *PR-2* activity to facilitate its passage through plant cells^25^. Moreover, a lack of tobacco *PR-2* lowers the risk of obtaining a virus^26^, whereas an overexpression increases the rate at which PVY infection spreads across cells^27^. Phytoalexins and PR proteins are known to influence plant disease resistance. Plant secondary metabolites, known as phytoalexins, are created in response to various stresses, primarily via the phenylpropanoid and terpenoid pathways^28^. Phenylalanine ammonia-lyase is responsible for catalyzing the initial steps in these two routes. Additionally, the *PAL* gene mediates the regulation of SA and JA in plants. Its expression kinetics support earlier research that found a link between viral infections and decreased *PAL* activity^29^. SA homeostasis directly influences its biological functions. Although a little quantity of SA produced by plants remains free, the majority of it undergoes a series of complex biological modifications such as glucosylation, methylation, amino acid conjugation, and hydroxylation ^30^. In plants, SA is rapidly metabolized to salicylic acid 2-O-β-D-glucoside and salicylic acid glucose ester for transport and storage. There are evidence that free and glycosylated forms modulate the immune responses in Arabidopsis, tomato, and cucumber^31^.

It was observed that once tomatoes were infected with TMV, the *HQT* gene expression rapidly fluctuated between up- and down-regulated. The main ways that polyphenolic compounds are made are through the chlorogenic acid (CHA), flavonoid, and phenylpropanoid pathways^32^. *HQT*, an essential enzyme, enhances CHA synthesis in plant tissues^33^. The RT-qPCR results showed that TMV infection lowered the *HQT* gene at 6 and 10 dpi. In addition, it was revealed that CHA has an essential role in enhancing plant disease resistance^34^. Consequently, severe symptoms could result from a TMV infection that successfully inhibits tomato plant CHA production. *CHS* is the first important enzyme in biosynthesis of flavonoids. In many plants, it changes p-coumaroyl CoA into naringenin chalcones^35^. When TMV infects tissues at 6 and 10 dpi, the expression of CHS was reduced. This shows that the virus suppresses the production of naringenin chalcones, especially in the early stages of the infection. This is linked to the fact that there was no naringenin in the HPLC analysis at 6 dpi. At 14 dpi, the level of expression started to increase again, and the presence of naringenin at a concentration of 6.33 µg/mL confirms this. According to phytochemical analysis using GC–MS and HPLC conducted on the tomato plants in comparison to mock plants, numerous phenolic, flavonoid, fatty acid, methyl ester, and sterol compounds were found in tomato plants during TMV infection at 6 and 14 dpi.

It was noted that three phenolic acids (*p*-coumaric, isoferulic, and ferulic) emerged in plants with TMV infection at 6 and 14 dpi. Ferulic acid (FA) is a naturally occurring phytochemical that is frequently found in leaves and seeds. It is known to be covalently conjugated to lignin, glycoproteins, polysaccharides, and polyamines. FA functions as an antioxidant, antibacterial, and antiviral, in addition to being critical for the cell wall's rigidity^36^. Furthermore, pyrogallol showed the highest levels in TMV-infected extracts only at 6 dpi and was significantly reduced at 14 dpi, indicating that TMV infection may suppress phenolic acids, which are produced in the greatest quantities by infected plant cells.

By contrast, caffeic acid exhibited the highest concentration in TMV-infected extracts at 14 dpi rather than 6 dpi. Caffeic acid is a hydroxycinnamic acid with many acrylic functional groups derived from plants. Because of its strong antioxidant action, it helps plants tolerate stress efficiently. It contributes in the control of pathogen attacks by fungi, bacteria, and viruses, as well as the management of salinity, ion toxicity, drought, and heavy metal stress^37^. These results are the same as those of Anuradha, who said that infecting *Passiflora edulis* fruit with the telosma mosaic virus increased the total amount of phenolics in the fruit^38^. It has actually long been recognized that polyphenol oxidation contributes to resistance to plant viruses such as TMV^39^. The increase in total phenolic content also revealed responses to numerous diseases, including pepper leaf curl virus disease^40^, Fusarium wilt^41^, tomato yellow leaf curl virus^42^, and pepper yellow mosaic virus disease^43^. Following a CMV infection, tomato plants also had higher levels of phenols, which promoted the lignification of cell walls and enhanced the plant's defenses against disease^44^. In the same way, members of the *Passifloraceae* family showed a 58.3% rise in secondary metabolites like flavonoids and polyphenols after being infected with a virus^45^. Meanwhile, it was shown that in TMV-infected tomato, several phenolic compounds (catechin, syringenic, cinnamic, salicylic, ellagic acid, and protocatchuic) were significantly reduced at 6 and 14 dpi compared to the control, demonstrating that TMV infection may inhibit the production of phenolic acids by infected plant cells.

HPLC analysis revealed that different flavonoid compounds were produced by TMV-infected tomatoes. Certain compounds exhibit an increase in concentration at 6 dpi, while others, such as naringin, rutin, flavone, and myricetin, are synthesized at 14 dpi. Even though three compounds (hisperdin, querestin, and chrysoeriol) were down-regulated following TMV infection, others were significantly reduced completely after TMV infection. This highlights the importance of spraying these compounds externally to increase plant resistance to TMV. Grapevine leafroll-associated virus-3-infected leaves exhibited a notable rise in flavonoid levels^46^. These results indicate that flavonoids are involved in the physiological responses to viral infections. According to Lou, CMV infection enhanced the expression of genes involved in flavonoid production in *Luffa cylindrical* L^47^. The two fatty acids acids, n-hexadecanoic and oleic, were found in large amounts in infected tomato leaf extracts at 6 dpi. These two chemicals' concentrations started to drop at 14 dpi. Both n-hexadecanoic acid and oleic acid have antibacterial properties^4849^. Additionally, it appears that saturated or unsaturated fatty acids may not have an impact on the replication of plant viruses in plant cells. Increasing the transcription of key genes in the phenylpropanoid pathway is known to change how flavonoids and phenolic compounds are made in tomatoes that have been infected with TMV. This helps the plant make compounds that protect itself. Furthermore, our findings imply that applying phenolic spraying at the initial stages of TMV infection may strengthen the tomato host's defense mechanism against the viral infection. Nevertheless, further investigation is necessary to comprehensively comprehend these viral pathogenic mechanisms. However, additional research should investigate the quantitative levels of salicylic acid, as well as the concentrations of its free and glycosylated forms.

## Materials and methods

### Isolation of tobacco mosaic virus

A tomato (*Solanum lycopersicum L*.) plant exhibiting TMV-like symptoms was collected from the open fields of New Borg El-Arab City, Egypt. Samples were transferred to the lab and directly analyzed for TMV infection using DAS-ELISA (DSMZ No.: RT-1065) following Clark and Adams procedure^50^. The tomato leaves infected with TMV were ground into a powder using a mortar and pestle in a solution containing 1-part leaves to 10 parts 0.1 M sodium phosphate buffer at pH 7.0, with the addition of 0.5% 2-mercaptoethanol. *Nicotiana glutinosa* plants were used as a local lesion host for TMV by lightly dusting them with carborundum (600 mesh) and gently rubbing them with a finger dipped in the freshly prepared inoculum^51^. Local lesions that appeared on the leaves of *N. glutinosa* four to five days after viral inoculation were used to get a pure viral isolate for inoculating tomatoes and further purification.

### Ethical approval

The authors confirm that the utilization of plants in the current research adheres to the appropriate institutional, national, and international guidelines and legislation. We obtained the necessary rights to collect plant specimens from the garden authorities and/or owners.

### Purification of TMV and transmission *electron* microscopy

The purification process began with inoculating tomato plants with a single local lesion from infected *N. glutinosa*, and after 20 days of viral infection, all symptomatic tomato leaves were collected for viral purification. TMV was purified by using polyethylene glycol (PEG) precipitation method^52^. The purified virus concentrations were determined by UV spectrophotometrically in at wave lengths 260 and 280 nm. Nickel grids coated with formvar were immersed in pure viral preparations for 5 min for TEM analysis. The grids were rinsed with distilled water and then stained with 2% phosphotungstic acid at pH 7.0 before being observed under a TMV (JEM-1400—Jeol Ltd., Tokyo, Japan).

### Molecular characterization of TMV

Following the instructions provided by the manufacturer (Geneaid Biotech, Taiwan), viral RNA was isolated from purified virus solution using a Plant Virus RNA Kit PVR050. First-strand cDNA was generated using a Thermo Scientific™ Revert Aid ™ first-strand cDNA kit (Thermo Scientific, USA)^53^. A specific primer target to the *TMV-CP* gene (Table 2) was used to amplify a product of 650 bp^54^. The PCR program consisted of an initial denaturation step at 95 °C for 3 min, followed by 35 cycles of denaturation at 94 °C for 1 min, annealing at 54 °C for 1 min, and extension at 72 °C for 1 min. A final extension was conducted at 72 °C for 5 min. Then, 5 µL of PCR products were separated by electrophoresis on a 2% agarose gel. The results were checked using a gel documentation system. The amplified products were purified using a PCR clean-up kit from Maxim Biotech Inc., USA, following the manufacturer's instructions. The purified amplicons were then sequenced. The annotated sequences were submitted to the GenBank database. The phylogenetic tree was generated by CLUSTAL W in the MEGA version 11 software^55^.

**Table 2:** Primer sequences utilized in this investigation.

Primer Name	Primer Code	Direction	Nucleotide Sequences (5’-3′)
Tobacco mosaic virus-coat protein	*TMV-CP*	Forward	ATTTAAGTGGASGGAAAAVCACT
		Reverse	CGGTCAGTGCCGAACAAGAA
Phenylalanine Ammonia-Lyase	*PAL*	Forward	ACGGGTTGCCATCTAATCTGACA
		Reverse	CGAGCAATAAGAAGCCATCGCAAT
Hydroxycinnamoyl Co A: quinate hydroxycinnamoyl transferase	*HQT*	Forward	CCCAATGGCTGGAAGATTAGCTA
		Reverse	ATGAATCACTTTCAGCCTCAACAA
Chalcone Synthase	*CHS*	Forward	CACCGTGGAGGAGTATCGTAAGGC
		Reverse	TGATCAACACAGTTGGAAGGCG
Pathogenesis related protein-1	*PR-1*	Forward	GTTCCTCCTTGCCACCTTC
		Reverse	TATGCACCCCCAGCATAGTT
Endoglucanase	*PR-2*	Forward	ATAGCCGTTGGAAACGAAG
		Reverse	CAACTTGCCATCACATTCTG
Elongation Factor 1-alpha	*EF1-α*	Forward	ATTGGAAATGGATATGCTCCA
		Reverse	TCCTTACCTGAACGCCTGTCA
Beta-actin	*β-actin*	Forward	GGCATACAAAGACAGGACAGCCT
		Reverse	CTCAATCCCAAGGCCAACAGAGA

### Greenhouse experimental design and sample collection

The current study utilized virus-free seeds of tomato cultivar G12 (*Solanum lycopersicum* L.). The seeds were sterilized and then cultivated in sterile vermiculite pots. The seedlings were transferred to new pots after twenty-eight days of germination. The sand and clay were mixed in a 1:1 ratio, and each 20 cm pot held 2 kg of the sterile mixture. A week later, the top two true leaves of each plant were mechanically inoculated with 0.5 mL of 10 µg/mL freshly prepared TMV. Ten pots with three plants each were used for the experiment. TMV was inoculated in six pots, and the rest were used as controls. A control group consisted of plants infected with buffer only (mock). The tomato leaves were collected from young leaves every two days on days 2–14 after TMV inoculation. The plants were cultivated in an insect-proof greenhouse with controlled conditions: 28 °C during the day, 16 °C at night, and 80% relative humidity. A 16-h sunshine cycle was maintained.

### Real-time quantitative PCR (RT-qPCR) for evaluation of defense responses

RNA was extracted from all samples using the GeneJET Plant RNA Purification Mini Kit (Thermo Scientific, USA), following the manufacturer's instructions. The LunaScript RT SuperMix Kit (New England Biolabs) was then used to synthesize cDNA, following the manufacturer's instructions. Reaction mixtures were carried out using 5 μL of RNA (1 μg), 4 μL LunaScript RT SuperMix, and 11 μL of nuclease-free water. The mixture underwent a 5 min incubation at 25 °C and a 10 min incubation at 45 °C. To stop the reaction, the PCR thermocycler (PEQLAB peqSTAR, Thermal Cycler 96 Universal Gradient Well, USA) was heated to 95 °C for 1 min. The resulting cDNA was stored at -80 °C. RT-qPCR reactions were carried out using an Applied Biosystems StepOne™ instrument thermal cycler (Applied Biosystems) and Luna Universal qPCR Master Mix (New England Biolabs). Following the manufacturer’s instructions for each 25-uL reaction volume, 1 μL of diluted cDNA was used as a template. Various primers were utilized to identify the expression of PR and polyphenolic-related genes, as outlined in Table 2. Each sample was assayed in triplicate. The ABI 7500 real-time PCR system was used as follows: 95°C for 10 min, then 40 cycles of 95°C for 15 s and 60 °C for 30 s, followed by 72 °C for 30 s. Transcript levels were standardized to two housekeeping genes of *β-actin* and *EF1-α* (Table 2). A melting curve (55–95 °C with 0.5°C increments) was created for each reaction to confirm the specificity of the PCR amplicons. The relative expression ratio was precisely quantified and estimated using the 2^-∆∆Ct^ algorithm^56^.

### Ethanol extract preparation and HPLC analysis

The tomato leaves from each group were collected, dried in the air, and pulverized into a fine powder using a mechanical blender. Two grams of leaves were extracted in 15 mL of 99% ethanol at 40 °C in a water bath for 5 h. The solution was filtered with Whatman No. 1 filter paper, transferred to a fresh tube, and then condensed using a rotary evaporator. The phenolic and flavonoid compounds were analyzed using an HPLC apparatus (Agilent Series 1100) from Agilent, USA. The apparatus consisted of an auto-sampling injector, solvent degasser, two LC pumps (series 1100), ChemStation version B.04.03 (https://www.agilent.com/en/product/software-informatics/analytical-software-suite/chromatography-data-systems/openlab-chemstation), and a UV/Vis detector set at 250 nm for phenolic acids and 360 nm for flavonoids. The analysis was conducted using a C18 column with dimensions of 125 mm × 4.60 mm and a particle size of 5 µm. The separation of phenolic acids was achieved by utilizing a gradient mobile phase consisting of two solvents: solvent A (methanol) and solvent B (acetic acid) in water at a ratio of 1:25. The gradient program started with a concentration of 100% B and remained at this level for the first three min. Subsequently, a 50% solution of eluent A was used for 5 min. Then, the concentration of A was raised to 80% for 2 min and subsequently lowered back to 50% for the next 5 min. The detection wavelength used was 250 nm. The separation of flavonoids was achieved by using a mobile phase consisting of two solvents: acetonitrile (A) and 0.2% (v/v) aqueous formic acid (B), with an isocratic elution program of 70:30. The solvent was flowing at a rate of 1 mL/min, and the separation process took place at a temperature of 25°C. The volumes of the injections were 25 μL^57,58^. Each sample was tested three times to verify accuracy. A standard group of thirteen compounds was used to find the phenolic compounds: cinnamic, ellagic, ferulic, gallic, isoferulic, *p*-coumaric, *p*-hydroxybenzoic, protocatchuic, pyrogallol, salicylic, and syringenic. To figure out the amount of flavonoid compounds, 7-OH flavone, catechin, chrysoeriol, hisperdin, kampferol, luteolin, myricetin, naringin, querestin, and rutin were used as standards.

### GC–MS analysis

The tomato leaves from each treatment were immersed in ethyl acetate in a 1:1 volume ratio for 20 min with shaking to extract the chemicals for GC–MS analysis. Subsequently, the solvent was subjected to evaporation. We used a Trace GC-TSQ mass spectrometer (Thermo Scientific, Austin, TX, USA) with a direct capillary column TG-5MS (30 m × 0.25 mm × 0.25 µm film thickness) to look at the chemicals in each sample. The temperature of the column oven was originally set at 50°C and then raised at a rate of 5 °C per min until it reached 250 °C, where it was maintained for 2 min. Subsequently, the temperature was increased to the final value of 300 °C at a rate of 30 °C per min and kept for an additional 2 min. The temperatures of the injector and MS transfer line were maintained at 270 °C and 260 °C, respectively. Helium was utilized as a carrier gas with a constant flow rate of 1 mL/min. The solvent delay was 4 min, and diluted samples of 1 µL were automatically injected using the Autosampler AS1300, which was connected to the GC in split mode. Electron impact (EI) mass spectra were obtained using an ionization voltage of 70 eV. The spectra were collected over the m/z range of 50–650 using full scan mode. The temperature of the ion source was adjusted to 200 °C. Each sample was tested three times to verify accuracy. The components were identified by comparing their mass spectra with those in the WILEY 09 and NIST 14 mass spectral databases^59,60^.

### Statistical analysis

The acquired data were statistically analyzed using a one-way analysis of variance (ANOVA) with a significance level of p ≤ 0.05 in the CoStat software version 6.311 (http://www.cohort.com). Statistically significant changes were demonstrated by analyzing the standard deviations (SD) of the relative gene expression levels. Transcriptional values above 1 indicate up-regulation, whereas values below 1 indicate down-regulation.

### Supplementary Information

Supplementary Information.

## Data Availability

The generated and analyzed data during the current study are included within the article and its Supplementary information as well as available from the corresponding author on reasonable request.
