# Supplementary material for: Transcriptional responses and secondary metabolites variation of tomato plant in response to tobacco mosaic virus infestation
Source: Sci Rep. 2024 Aug 22;14:19565. doi: 10.1038/s41598-024-69492-3 (PMC11341961; doi:10.1038/s41598-024-69492-3)
Supplement: Supplementary file 1 — Supplementary Information. [file 41598_2024_69492_MOESM1_ESM.pdf]

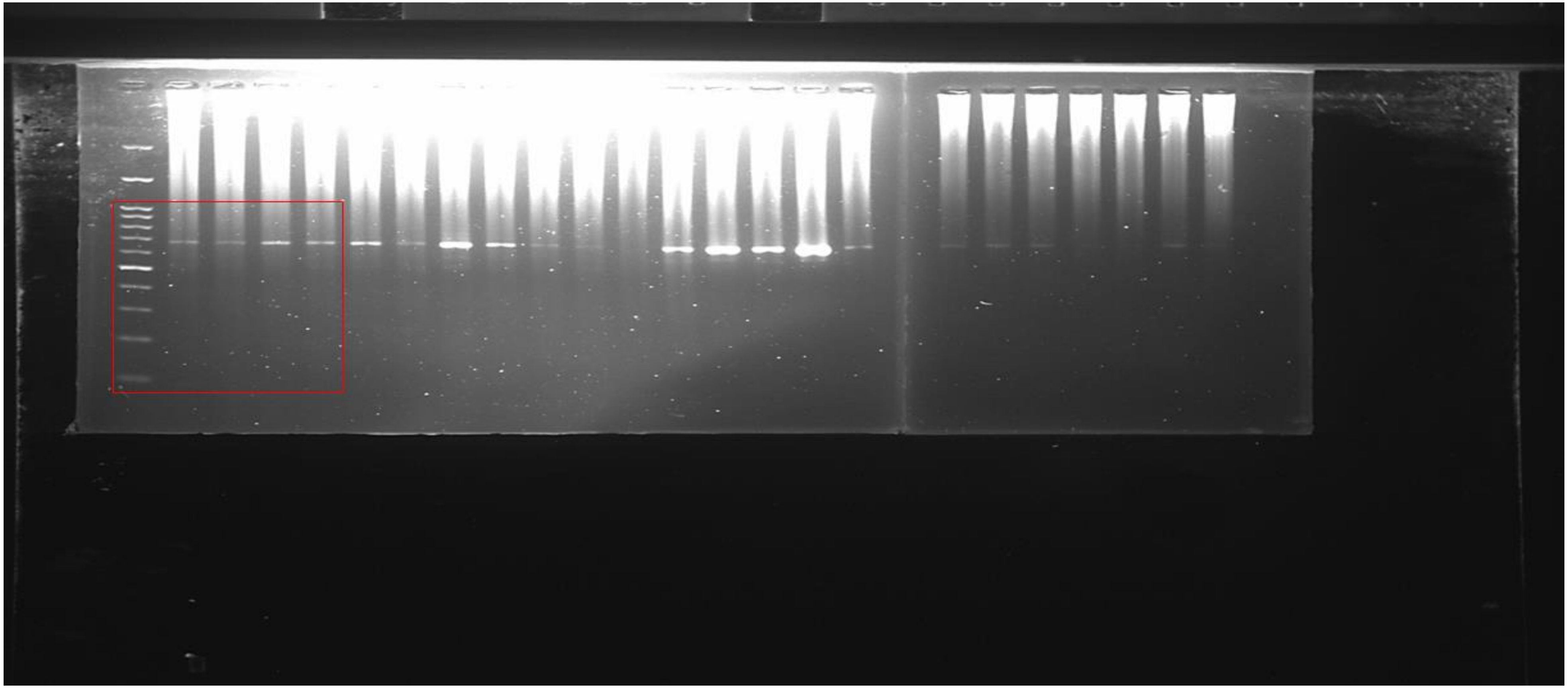

Figure S1. Gel electrophoresis image of RT-PCR amplification of the TMV-CP gene from different tested samples developed local lesions on TMV-infected *Nicotiana glutinosa*. The red square presented the cut and presented image in the manuscript
